# Secondary Prevention of Rheumatic Heart Disease in Nepal: Are We Going Backward?

**DOI:** 10.31729/jnma.7861

**Published:** 2022-09-30

**Authors:** Prakash Raj Regmi, Mandip Dhungel, Riju Kafle

**Affiliations:** 1 Nepal Heart Foundation, Pulchowk, Lalitpur, Nepal; 2 HAMS Hospital, Mandikhatar Road, Kathmandu, Nepal

**Keywords:** *benzathine penicillin*, *rheumatic heart disease*, *secondary prevention*

## Abstract

A secondary level of prophylaxis has proven to be the most successful in Nepal, a country with an endemic rate of rheumatic heart disease, in combating the severe issues associated with rheumatic heart disease. The use of benzathine penicillin G in secondary prophylaxis of rheumatic heart disease, recommended by several guidelines, has been increasingly abandoned in Nepal due to a lack of national guidelines and the termination of the prior programs. The use of oral penicillin and alternative oral antibiotics, which are less effective in preventing the recurrence of acute rheumatic fever, is on the rise. Nepal urgently needs to develop new national guidelines and ensure their effective implementation in order to slow the increase in the number of rheumatic heart disease patients. In this article, we explore the limitations, challenges, and advantages of using the consensus-supported intramuscular benzathine penicillin G as the first-line drug for the secondary prevention of rheumatic heart disease.

## INTRODUCTION

Rheumatic Heart Disease (RHD) is a late sequence of Rheumatic Fever (RF), which develops years or even decades after the initial insult. It poses a significant health burden in developing countries, being the most common cardiovascular disease in individuals aged <25 years.^1,2^ To combat this public health problem, there has been an expert consensus to prevent the disease with three levels of prevention.^3^

Secondary level of prevention is inexpensive and feasible even in remote places to prevent the disease from taking a catastrophic role.^4,5^ If the secondary levels of prevention are left unchecked, it can overwhelm an already overburdened tertiary setting.^6^

## RHEUMATIC HEART DISEASE PROGRAM IN NEPAL SO FAR

Due to a lack of early intervention and an overwhelmed tertiary system, young people have died awaiting heart valve replacement. Thus, in 2007 AD the Nepal Heart Foundation (NHF), with funding from the government of Nepal (GoN), developed a comprehensive program of community awareness, free medication, RHD register development, health worker training, guideline development, and clinical audit to tackle the growing burden of RHD. The key focus of the program had been to deliver antibiotics for the secondary prophylaxis of RHD.^7^ However, the initial RF/RHD prevention and control program was halted due to a lack of sufficient funds and the government of Nepal directed its resources toward conducting free-of-cost heart valve surgeries.^8^

Owing to a lack of proper guidelines and no system for implementation of secondary prophylaxis, there has been a decrease in penicillin use, with a low focus on injectable penicillin. In a study conducted in Bharatpur, it was observed that 73.33% of the patients receiving secondary prophylaxis had received oral penicillin and only 21.90% of the patients had received intramuscular benzathine penicillin, the ratio being 3.35:1.^4^

## SECONDARY PREVENTION OF RHEUMATIC HEART DISEASE

There are ample pieces of evidence, including a metaanalysis, to support the use of penicillin as the most effective secondary method of prophylaxis for RHD. Moreover, intramuscular benzathine penicillin G (BPG) is more effective than oral penicillin in the secondary prevention of acute rheumatic fever (ARF), and is, therefore, a first-line drug for the secondary prevention of ARF and RHD.^9-12^ It is also more effective than long-acting azithromycin, erythromycin, or sulphonamide.^13^

Various country-based and organization-based guidelines such as the American Heart Association (AHA) guidelines,^10^ World Health Organization (WHO), New Zealand Guidelines for Rheumatic Fever, Indian Academy of Paediatrics, Australian guideline for prevention, diagnosis, and management of acute rheumatic fever and rheumatic heart disease, South African National Guidelines on Primary Prevention and Prophylaxis of RF and RHD for Health Professional at Primary Level, and Saudi Pediatric Infectious Diseases Society (SPIDS) also recommend intramuscular BPG as the medication of choice scheduled to be administered every 4 weeks depending on the patients. Most of the aforementioned guidelines recommend treatment with oral penicillin V or sulfonamides as an alternative regimen in patients with ARF and RHD and with erythromycin in patients allergic to penicillin.^14^

## RETURN OF WHO IN RHD PREVENTION ACTIVITIES

In the World Health Organization (WHO)'s Global Action Plan for the Prevention and Control of Non-Communicable Diseases 2013-2020, one of the policy choices for the Member States is the secondary prevention of rheumatic fever and rheumatic heart disease. The majority of deaths caused by rheumatic heart disease are avoidable, and eradicating it will make us one step closer to achieving the global objective of reducing premature mortality from non-communicable illnesses by one-third by the year 2030.

The WHO global initiative for the prevention and control of RHD was primarily focused on secondary prevention which included active case finding, registry maintenance, secondary prophylaxis, training, and health education. This initiative which lasted from 1984 to 2000 was proven to be highly effective in the reduction of disease burden in endemic countries like India, China, Cuba, Egypt, and the Philippines. WHO urges its member states and its secretariat to again undertake various actions directed against RHD as stated in the report by the Director-General.^15^

## CHALLENGES TO SECONDARY LEVEL OF PREVENTION IN NEPAL AND SOME SOLUTIONS

### LACK OF GUIDELINES

The challenges of delivering quality health care are enormous when there is no national guideline. Nepal is also currently without a system in place to tackle the challenges related to intramuscular benzathine penicillin planning, storage, and logistics. Therefore, the first-line secondary prophylaxis with intramuscular BPG has been decreasing over the last decade.^4^

The World Heart Federation (WHF) which has a goal of a 25% reduction in premature deaths from RF and RHD among individuals aged <25 years by the year 2025, provides the foundation for governments, civil society, patient advocates, clinicians, researchers, and funding agencies to develop partnerships and unify global efforts to control RF and RHD.^2^

### LOW COMPLIANCE

Another challenge that is commonly faced is the lower compliance of intramuscular BPG administration due to fear of allergy, local site pain, and long duration of treatment.^13,16^ During secondary prophylaxis for delivering BPG in the RF/RHD Program of Nepal previously, paramedics were given training and support for injection BPG administration. Around 90% of paramedics who had earlier had misgivings about injecting BPG due to fear of allergic events, had been happy to do so after effective training.^7^

### LACK OF YOUTH INVOLVEMENT

The secondary prophylaxis program is mostly targeted at adolescents and young adults who can find the treatment unpleasant. Therefore, it is necessary to make this program adolescent-friendly through the use of social media, involving youths in the community awareness programs, and increasing their involvement in RHD policy and decision making. It is important to focus on the importance of receiving prophylaxis injections and the potential for minor throat illness and joint pain to escalate into serious heart disease years later.^17^

### LACK OF ADHERENCE

Another way to increase adherence is by keeping a community-level nursing register with updated patient information, contacts, and injection data. Digitizing this system could further benefit patients whose injection status can be updated through the internet in real-time, significantly improving compliance.^13,17-19^ RF/RHD register contains the list of people who have an established diagnosis of RF/RHD with brief details. These registers bridge the gap that an unstructured program creates like missing out on doses of BPG. The use of these registries came into existence in The US in the 1950s and made the distribution of novel therapies efficient in reducing the disease burden.^20^ Similarly in a study that included a young population in New Zealand, utilization of registry-based care resulted in receiving 94% of their secondary prophylaxis as compared to those who received only 37% through an unstructured primary care program.^21^ The RHD register aids in the proper distribution of secondary prophylaxis but its usefulness extends to the development of a priority-based comprehensive follow-up program. The clinical demands of the patients at varying stages of the disease differ, and a system is required to ensure that those who require the greatest assistance receive it. One answer to this challenge is to assign priorities to distinct groups of patients. A method of "priority-based follow-up" provides a framework for scheduling and organizing follow-ups.^14^

### ADVERSE EFFECTS OF BPG

Antibiotics including penicillin can cause hypersensitivity reactions and adverse drug reactions (ADRs) via a number of different mechanisms. Anaphylaxis may result in the death of the patient.^22^ The most prominent adverse reaction of intramuscular BPG is the sharp pain in the injection site and its intensity following administration of BPG may decrease compliance with the regimen. Therefore, improvement in tolerability may be required to maintain adherence.^23^ It is imperative that long-acting prophylactic penicillin administration methods be improved for the secondary prevention of RHD for example,^24^ using Z-track techniques and internally rotated foot,^25^ BPG diluted with anesthetic significantly reduces the pain of IM administration of BPG,^26^ etc. For preventing deaths from anaphylaxis, injection delivery safety measures recommended by NHF should be widely practiced.^6^

### LACK OF AVAILABILITY

The complicated tiers of the manufacturing process with the active pharmaceutical ingredient (API) produced by the primary manufacturers and then selling the API to secondary companies for packaging, distribution, and sale prolong the manufacturing time. Although the precise number has not been determined, it is assumed that a challenging supply chain and slim profit margins have reduced the number of API manufacturers.^27^ Delivery delays and stockouts have also been caused by pharmaceutical companies' diminished commercial incentive to produce BPG.^28^ WHO has added BPG in every edition of its Essential Medicine list. Hence, BPG is anticipated to be readily available in the majority of low- and middle-income countries where RHD is endemic.^29^

## WAY FORWARD

There is a global consensus that intramuscular BPG is the first-line drug for secondary prophylaxis for RHD. Thus, the downward trend of its use in Nepal is concerning. A clear national guideline about the drugs for secondary prophylaxis, dose, dosing frequency, and duration needs to be outlined. It is our collective responsibility to implement the guidelines, from the government level to the grassroots level. A united front to implement the most effective secondary level of prevention of RHD will change the face of RHD incidence in Nepal. Understanding the challenges for the adherence and compliance of intramuscular BPG, and outlining the solution to those will be necessary for the secondary level of prevention to be a success in Nepal.
